# Fetal and Neonatal Levels of Omega-3: Effects on Neurodevelopment, Nutrition, and Growth

**DOI:** 10.1100/2012/202473

**Published:** 2012-10-17

**Authors:** Juliana Rombaldi Bernardi, Renata de Souza Escobar, Charles Francisco Ferreira, Patrícia Pelufo Silveira

**Affiliations:** Núcleo de Estudos da Saúde da Criança e do Adolescente, Hospital de Clínicas de Porto Alegre (HCPA), Universidade Federal do Rio Grande do Sul, Rua Ramiro Barcelos 2350, 90035-903, Porto Alegre, RS, Brazil

## Abstract

Nutrition in pregnancy, during lactation, childhood, and later stages has a fundamental influence on overall development. There is a growing research interest on the role of key dietary nutrients in fetal health. Omega-3 polyunsaturated fatty acids (n-3 LCPUFAs) play an important role in brain development and function. Evidence from animal models of dietary n-3 LCPUFAs deficiency suggests that these fatty acids promote early brain development and regulate behavioral and neurochemical aspects related to mood disorders (stress responses, depression, and aggression and growth, memory, and cognitive functions). Preclinical and clinical studies suggest the role of n-3 LCPUFAs on neurodevelopment and growth. n-3 LCPUFAs may be an effective adjunctive factor for neural development, growth, and cognitive development, but further large-scale, well-controlled trials and preclinical studies are needed to examine its clinical mechanisms and possible benefits. The present paper discusses the use of n-3 LCPUFAs during different developmental stages and the investigation of different sources of consumption. The paper summarizes the role of n-3 LCPUFAs levels during critical periods and their effects on the children's neurodevelopment, nutrition, and growth.

## 1. Introduction

Arachidonic acid (AA; 20:4n-6) and Docosahexaenoic acid (DHA; 22:5n-3) are essential for brain growth and cognitive development; they also accumulate rapidly in the brain and retina during the later stages of gestation and early postnatal life [1–3].

During pregnancy, AA and DHA are transported across the placenta into fetal venous blood [4, 5]. Nutrition in pregnancy has a significant influence on fetal development. In this sense, there is an increasing interest on the role of key dietary nutrients in subsequent health status or diseases [6]. The major effects of higher levels of omega-3 polyunsaturated fatty acids (n-3 LCPUFAs) during pregnancy are associated with the development of brain and nerve tissues during the period of maximum intrauterine accretion [7].

After birth, breast milk is the source of AA and DHA to the neonate; the content of essential polyunsaturated fatty acids (PUFAs) in breast milk is dependent on the maternal intake of these fatty acids [8, 9]. The content of essential PUFAs may be low in populations eating primarily plant-based diets with no or limited fish intake [10]. The consumption of PUFAs is essential for the development of the brain and nervous system of children and teenagers [11].

The contribution of DHA for better neurodevelopment has been documented in breastfed infants. However, the effects of different levels of DHA in human milk and maternal DHA supplementation limited to the lactation period are still under investigation [7]. In foods, the most important source of DHA is fish and fish oil [12]. Sea fish are generally a better source of DHA than freshwater fish [10]. Hence, in many studies the consumption of marine foods is evaluated separately.

Researchers and mothers have turned to fish oil supplements, which are generally low in contaminants (methylmercury) and can provide substantially higher doses of n-3 LCPUFAs than most adults are able to consume in their diets [13]. In a recent review of studies in 13 countries within the lower and middle worldwide brackets of gross domestic product (GDP), there was a strong positive association between the economic status of the country (GDP) and the supply of total fat and n-3 LCPUFAs. In families living on a primarily plant-based diet, most of the fat and PUFAs intake come from vegetable oils and cereals [10].

It is evident that several worldwide health agencies recognize the importance of increasing dietary intake of n-3 LCPUFAs. The daily DHA recommendation during pregnancy from the Expert Committee ranges from 200–300 mg/daily (modified from [14]). For the first 6 months of life of preterm and term infants, dietary intakes of n-3 LCPUFAs should correspond to 0.45–1.5 energy% of total of fatty acids, and with an n-6 LCPUFAs to n-3 LCPUFAs ratio of 4–10 (modified from [15]). The Institute of Medicine (IOM) recommends a range of 0.6–1.2 energy% in n-3 LCPUFAs and a range of 5–10 energy% in n-6 PUFAs in children and adults [16].

In the present paper, we review the scientific evidence that relates n-3 LCPUFAs levels during these critical periods to children's neurodevelopment, nutrition, and growth. PubMed database was used to identify relevant studies, and reference lists from retrieved studies were reviewed. Study selections were limited to English and Portuguese studies involving humans and experimental studies, from 1980 to 2012.

## 2. Dietary Intake and Total Fat Status

In Iran, authors found a significant difference in the total n-3 LCPUFAs and DHA in breast milk in women in coastal cities compared with those in inland cities. Coastal mothers showed a significantly higher intake of fish or seafood compared with inland mothers (*P* < 0.001). The dietary intake of fish and seafood was assessed using a structured interview, called food frequency questionnaire (FFQ). Breast milk was collected from the mother's breasts by hand, pumping eight to 72 h after delivery. The study showed that dietary fish and seafood intake by the mother influenced the DHA content of breast milk.

A randomized controlled trial investigated direct supplementation of high dose fish oil to term infants in the early postnatal period (with regard to feeding practices). Infants were supplemented daily, from birth to six months, with fish oil supplement or placebo. The results indicate that direct fish oil supplementation can significantly elevate DHA levels in infancy. The study showed a successful strategy for raising infant DHA status [18]. In breastfed infants, DHA levels in breast milk were determinant for their DHA status [18].

## 3. Impact of Fatty Acids Status on Neurobehavioral Development

Long-chain polyunsaturated fatty acids (LCPUFAs) play an important role in the central nervous system (CNS) and are essential for early brain development. LCPUFAs comprise approximately 15 to 30% of the brain's dry weight [19]. LCPUFAs are involved in the functioning and architecture of the central nervous system; the function of LCPUFA is demonstrated, in part, through the dependence on LCPUFA through different stages in life. The brain's structure is lipid and consists mainly of phosphoglycerides and cholesterol rich in AA and DHA [20]. Studies show that DHA affects blood-brain barrier functions and neuronal membrane fluidity; it also regulates neurotransmission systems such as serotonergic, dopaminergic, norepinephrinergic, and acetylcholinergic systems [21, 22]. DHA has a significant effect on neuronal membrane dynamics and therefore on transporter, receptor, and neurotransmitter functions [19].

The fastest stage of neural development is during fetal growth. However, there is also significant development during the first five years of postnatal life. At this time, environmental factors, including nutrient intake, play a critical role in the development of the brain's cytoarchitecture [23]. n-3 and n-6 LCPUFA are required for proper brain function and for the development of a mature brain. n-3 and n-6 LCPUFA are important due to their effects on electrophysiology and cell membrane structures [24]. Human milk fatty acid composition depends on short-term and long-term maternal diet [25]. The early diet can provide the proper amount of DHA and n*-*6 LCPUFA to the infant's brain [26]. The psychophysiology of n-3 LCPUFAs indicates the potential effects of n-3 LCPUFAs in relation to direct changes in the brain; the potential effects include changes in cognitive measures [27].

During the third trimester of pregnancy, fetuses require approximately 40 to 60 mg of n-3 LCPUFAs per kilogram of body weight per day [28]. During the last gestational trimester and first postnatal months, there is significant growth of the human brain and a large increase in cerebral volume of DHA and AA [29]. During prenatal and perinatal growth, significant amounts of AA and DHA accumulate in the brain and hence the physiological demand for these nutrients [28].

Studies have shown evidence for the neurodevelopmental benefits of dietary PUFA during pregnancy and infancy [27, 29, 30]. In addition to the neural tissue, the retinal tissue also accumulates LCPUFA DHA during gestation. In retina rod cells, DHA accounts for 50 to 60% of the fatty acids used in the polar phosphoglycerides of the plates [20, 22]. Preterm infants are denied the full intrauterine supply of DHA available to term infants [31]; therefore, preterm infants depend on a diet that supplies all the DHA needed for growth and development.

Several studies have shown the cognitive benefits of high levels of consumption of n-3 LCPUFA in elderly populations [32–34]. There is a link between EPA and the central nervous system, which has been shown in studies that indicate that n-3 LCPUFA levels are associated with protection against certain diseases including Alzheimer's and multiple sclerosis. In animal study, author showed that EPA stimulates the expression of myelin-related proteins in rat brains. Better myelination may be associated with improved segregation of electrochemical signals transmitted to the axons; this may lead to faster speed of information processing and improved cognition among people with high levels of n-3 LCPUFAs consumption [36].

### 3.1. Dietary Consumption: n-3 LCPUFAs

Reference [37] showed a positive association between DHA concentrations in breast milk and infant scores on the NBAS (Brazelton Neonatal Behavioral Assessment Scale) Range of State cluster score. The results suggest that DHA is related to infant's superior ability to maintain optimal arousal.

Maternal intake of n-3 LCPUFA and breastfeeding predicted oppositional defiant and conduct disorder and comorbid externalizing disorder, before adjustment for confounding factors. However, there was no association between intake of n-3 LCPUFA by the mother or child with any type of externalizing disorder once sociodemographic factors were taken into account [38]. Studies show inconsistent results about the effects of n-3 LCPUFA on externalizing disorders: some studies report positive effects of n-3 LCPUFA [39, 40], but others have shown less conclusive results [41]. 

Confounding factors associated with inherited factors such as maternal IQ [42] and maternal nutrition present serious challenges in these studies. Prenatal exposure may persist into the postnatal environment; therefore, the identification of the precise timing of factors poses a scientific challenge. Nutrition, of course, has an essential role in mental development during different life stages. Reference [43] showed that breastfeeding is associated with positive effects on cognitive development. However, the evidence about the parallel effects of breastfeeding on emotional development is inconsistent [38, 44, 45]. There are also nonnutritional reasons why breastfeeding may influence mental health, including closeness and reciprocity [46].

Reference [47], in a double-blind randomized controlled trial, investigated the effects of raising intakes of EPA and DHA on depressed mood in mild to moderately depressed individuals who were not receiving conventional treatments for depression. The results did not show a significant effect of n-3 LCPUFA supplementation on depressed moods. Participants in the intervention group received three daily doses of 630 mg EPA, 850 mg DHA, 870 mg olive oil, 7,5 mg mixed tocopherols, and 12 mg orange oil (which was included to improve palatability and blinding) for 12 weeks. The measures of depressed mood for the experimental group were not better than those for the placebo group. 

A review of studies that investigated the effects of n-3 LCPUFAs and mental illness found consistent evidence about the beneficial effects of n-3 LCPUFAs in relation to mood disorders. The results of studies that investigated an association between n-3 LCPUFAs and schizophrenia, borderline personality, attention deficit hyperactivity, and symptoms of anxiety are inconclusive. The results do not support indication of monotherapy or adjunctive treatment for mental illnesses [48].

However, there is evidence of n-3 LCPUFAs deficiency identified in women who develop postpartum depression. Postpartum depression is a serious mood disorder with the same symptoms as major depressive disorders (depressed mood, loss of interest in pleasurable activities, changes in sleep patterns and weight, and reduced cognitive abilities and concentration) [49, 50].

Observational studies show that prevalence of postpartum depression may be associated with n-3 LCPUFAs (FAs) intake and status. However, there are few controlled trials that have evaluated the effects of increasing the intakes of FAs on depression symptoms in postpartum women. Most interventions studies investigated a reduced number of patients and typically provided fish oil-based supplements that contained high doses of EPA and DHA [51]. Prenatal fish consumption was not predictive of postnatal depression (PND); also, postnatal n-3 status was not associated with postnatal depression. However, prenatal fish consumption predicted n-3 status in mothers after birth. Based on these findings, it is difficult to assess the efficacy of n-3 LCPUFAs for the treatment of PND [52].

### 3.2. Supplementation: n-3 LCPUFAs

A recent review showed unclear effects of maternal prenatal and postnatal LC-PUFAs supplementation on global neurobehavioral outcomes for children born to term. However, n-3 LCPUFAs supplementation for breastfeeding women of preterm infants improved performance on tests of global neurodevelopment [53].

Fish oil supplementation (4 g of fish oil with 1.1 g EPA and 2.2 DHA/daily) starting at 20 weeks of pregnancy until birth significantly increased the fatty acid composition of maternal and neonatal erythrocytes in comparison with olive oil supplementation. The effects were partially sustained until at least 6 weeks postpartum. Similarly, administration of n-6 polyunsaturated fatty acids AA was associated with significantly lower erythrocytes in neonates in the fish oil group in comparison with controls [54].

A DOMInO (DHA to Optimize Mother Infant Outcome) study with 2,399 women investigated whether DHA supplementation during the second half of gestation reduced the risk for depressed maternal mood (Edinburgh Postnatal Depression Scale) during the postpartum period. Women in the DHA group consumed 3 daily capsules of fish oil that contained a total of 800 mg DHA and 100 mg EPA (eicosapentaenoic acid); women in the control group consumed similar supplement levels of vegetable oil. The group given DHA did not show significant reduction in the rates of PND [55].

In the same study, researchers investigated early cognitive development in the offspring at 18 months of age. The results did not show an overall effect of DHA on development (assessed using the Bayley Scales of Infant and Toddler Development including cognitive scores and language scores [55]). With the objective of determining whether DHA deficiency occurs in pregnant women and contributes to poor infant development, 200 mg/g of DHA was administered to women from 16 weeks of gestation until delivery. The results showed a visual acuity below average among infants in the placebo than in the DHA intervention group [56].

Daily maternal supplementation with 1.6 g EPA or 1.1 g DHA from the 25th gestational week to an average 3-4 months in early breastfeeding decreased the risk for food allergies and IgE-associated eczema during the first year of life. The effects were shown in infants with a family history of allergic disease compared with a placebo group [57].

Supplementation with a relatively high dose of fish oil (1.1 g EPA and 2.2 g DHA/daily) during the last 20 weeks of pregnancy is not only safe but also has potential beneficial effects, but the effects need to be further investigated. At age 2.5 years, children in the fish oil-supplemented group showed a significantly higher score for hand-eye coordination than children in the placebo group. There was a correlation between hand-eye coordination scores with n-3 LCPUFAs levels in cord blood erythrocytes, and a negative correlation with n-6 PUFA [58].

Reference [29] found that giving pregnant and lactating women n-3 very long-chain PUFAs supplementation was associated with higher IQ scores at 4 years of age in comparison with maternal supplementation with n-6 long-chain PUFAs. There were no significant differences on the Kaufman Assessment Battery for Children test at 7 years of age between children whose mothers had taken cod liver oil (n-3 LCPUFAs rich) or corn oil (n*-*6 PUFA rich) [59].

A multicenter, randomized controlled trial from DINO (Docosahexaenoic Acid for the Improvement of Neurodevelopmental Outcome in Preterm Infants) with 657 lactating administered six 500 mg DHA-rich tuna oil capsules per day (for breast milk DHA concentration). The study showed that a DHA dose of approximately 1% total fatty acids in early life did not increase Bayley Mental Developmental (MDI) scores of preterm infants with less than 33 weeks of gestation. However, the dosage improved the MDI scores of girls [60].

In another study, breast-feeding women received identical capsules containing either DHA algal oil (200 mg/d of DHA) or a vegetable oil (no DHA) from delivery until 4 months postpartum. The results showed that at 5 years, there were no differences in visual function (Bailey-Lovie acuity chart), transient visual evoked potential, or sweep visual evoked potential testing between children whose mothers received DHA versus placebo. However, children whose mothers received DHA versus placebo performed significantly better on the Sustained Attention Subscale of the Leiter International Performance Scale. There were no statistically significant differences between groups on other neuropsychological domains [61].

Yet another study was carried out with the objective of determining the effect of DHA supplementation on neurodevelopment status and visual function. Breastfeeding mothers were administered capsules containing either high-DHA concentration algal oil (200 mg DHA/day) or a vegetable oil (no DHA) to breastfeeding women for 4 months after delivery. The intervention group showed 50% greater content of maternal plasma phospholipids DHA, 75% greater content of DHA in milk lipids, and 35% greater content of DHA in infant plasma phospholipids. However, higher infant plasma phospholipids DHA content did not show effects on visual acuity at either 4 or 8 months of age [62].

The DINO Trial DHA found that supplementation for infants <33 weeks gestation with tuna oil capsules reduced the incidence of bronchopulmonary dysplasia in boys and in all infants with a birth weight <1250 g; it also reduced the incidence of reported hay fever in boys at either 12 or 18 months [63].

Reference [64] compared the content of DHA in breast milk in three groups: mothers with habitual high fish intake, with fish oil supplementation and with olive oil supplementation. The authors described a small effect of DHA levels in breast milk on early language development (word production) of breast-fed infants. No beneficial effect was reported for problem solving, though the data suggest there may be an effect in girls.

Reference [65], in a follow-up study, evaluated IQ, receptive and expressive vocabulary, visual-motor function, and visual acuity in formula-fed and breast-fed infants. Healthy term infants (*n* = 197) less than a week old were randomized to be fed formulas for one year, one containing DHA or another containing both AA and DHA; there was also a group of infants who were exclusively breastfed for at least 3 months. At 39 months, IQ, receptive and expressive language, visual-motor function, and visual acuity were not different between the randomized formula groups or between the breastfed and formula groups. Growth achievement, red blood cell fatty acid levels, and morbidity did not differ between the groups [66].

### 3.3. Use of Food Supplements with n-3 LCPUFAs

In an observational study of subjects born in 1936 whose mental ability was tested in 1947 and who were followed up in 2000-2001, the childhood IQ did not differ significantly by category of food supplement use. However, at the age of 64 years, cognitive function was higher in food supplement users than in nonusers, before adjustment for childhood IQ [67]. In a nested case-control study, erythrocyte membrane n-3 content was higher in fish oil supplement users than in nonusers, but cognitive function did not differ significantly between the groups [67].

## 4. Impact of Fatty Acids Status on Nutrition and Growth

A recent review limited to human studies published in English from 2000 until 2010 on n-3 and growth suggests that n-3 (DHA in particular) during pregnancy, lactation and early life may be associated with significant benefits for infant growth and development in developing countries. Studies in developing countries show that a higher n-3 intake or supplementation during pregnancy may result in improvements in birth weight, length, and gestational age. Limited data from developing countries suggest that ALA or DHA supplementation during lactation and in infants may be beneficial for growth and development of young children; these benefits are more pronounced in undernourished children. However, there is no evidence of improvements in growth following n-3 supplementation in children >2 years of age [68].

### 4.1. Dietary Consumption of n-3 Fatty Acid

Reference [69] compared the levels of the main long-chain PUFA between birth and the first year of age. The results showed a significant decrease with age in the proportion of these fatty acids in the plasma of children. At one year of age, the percentage of DHA in the extended breastfeeding group (more than 6 months) differed significantly from the other groups, that is, medium term breastfeeding (more than 3 and less than 5 months) and exclusive formula feeding.

An interventional study carried out in Brazil showed that postnatal sardine consumption (100 g of sardines two or three times a week) contributed to an increase omega-3 series fatty acids (DHA and DPA) on the composition of breast milk of mothers at 15 and 30 days into the study [70].

In a prospective, observational cohort study with 2109 subjects who were enrolled in the Project Viva, in the USA, the authors investigated if maternal marine n-3 LCPUFAs and seafood intake were associated with birth weight, birthweight for gestational-age *z* value (fetal growth), length of gestation, low birth weight, preterm delivery, and babies small for gestational age. The study showed that increased consumption of the n-3 LCPUFAs DHA and EPA was associated with a modest decrease in fetal growth. The study did not show any association between higher seafood or n-3 LCPUFAs intake and length of gestation [71].

A prospective study with 62099 women in Norway (Norwegian Mother and Child Cohort Study-MoBa) investigated the influence of maternal intakes of seafood and supplementary n-3 LCPUFAs in infant birth weight, length, and head circumference. The researchers found that maternal seafood consumption was positively associated with birth size, driven by lean fish intake, while supplementary n-3 intake was negatively associated with infant head circumference [72].

### 4.2. Supplementation with n-3 LCPUFAs

#### 4.2.1. Experimental Studies

A study was conducted with 22 Lister Hooded rats that are divided into two groups: the control group (CG) which received a casein-based diet, and the flaxseed group (FG) which received 25% flaxseed diet supplement, and 14% casein showed that, during gestation and lactation, the FG was similar to the CG group in relation to food intake and litter size. The same result was observed for milk fat content and total energy value. At weaning, the FG group was similar to the CG group in both in offspring body weight and in weight gain of pups during lactation [73]. However, a study using only female Wistar rats (*n* = 16) and the same group division (FG and CG) showed that mothers on the flaxseed diet gained less weight during lactation and their offspring had lower body weight only after weaning. At 170 days, lower hemoglobin levels were observed in the FG group, but there was no statistically significant difference in visceral fat mass between groups [74].

A study with female Wistar rats (*n* = 18) administered three types of diet: control group (CG) with 7% soy oil; flaxseed group (FG) with 10% flaxseed diet (25 g), both fed ad libitum diets and a modified control group (MCG) with 10% soy oil, pair-fed with the FG. The diets were given to dams during preconception, pregnancy, and lactation and to their pups after weaning. The offspring of FG dams showed lower body mass than those of CG dams. The content of DHA in the hippocampus was higher in the FG, followed by the MCG compared with the CG. Hippocampal DHA content correlated with better spatial memory performance in the FG, whereas AA content correlated with longer time in solving tasks [75].

#### 4.2.2. Clinical Studies

A recent review shows that LC-PUFAs supplementation during pregnancy is associated with modest increases in birth size in both low-income and high-income populations. However, postnatal supplementation with LC-PUFAs did not influence infant growth [53].

Babies born of primiparous Mexican women who received 400 mg/d of algal DHA during the second half of the pregnancy term were heavier and had larger head circumferences at birth than children whose mothers received placebo (olive oil). However, no effect was observed in multiparous mothers. Children of primiparous women who were given DHA were taller at 18 months than those of primiparous women given placebo. The authors suggest that since primiparous women were, on average, younger than the multiparous women, their own body stores of DHA are not well established and available to the fetus and infant [76].

Reference [62] found that there were no statistically significant differences in weight, length, or head circumference between the 2 groups of infants at any time (high-DHA algal oil or a vegetable oil without DHA for 4 months after delivery). At all ages, these measures were within the normal range for age in both groups.

Supplementation with fish oil (1.1 g EPA and 2.2 DHA/daily) or olive oil starting at 18–20 weeks of pregnancy and continued until delivery showed similar growth measurements in the two groups at age 2^1/2^ years [58].

A randomized, double-blind trial gave lactating mothers fish oil or olive oil supplements for 4 months; the study followed infant development for 2.5 years and showed that children from the fish oil group had larger head circumference and higher BMI than those in the olive oil group after adjustment for relevant confounds. Both body composition and head circumference at 2.5 years of age positively correlated with the DHA content of maternal red blood cells at the end of the intervention [77].

## 5. Conclusions

Experimental, biochemical, and epidemiological evidence support the hypothesis that low n-3 polyunsaturated fatty acids (n-3 LCPUFA) levels are associated with dysfunctional brain development and impaired growth; the evidence also shows that n-3 supplementation is useful for improvement of specific parameters, especially those related to memory and cognition. However, the precise definition of the method of use and dosages is still unclear. Large-scale, well-controlled trials should be carried out to confirm the efficacy of the acids and establish the minimum dose and length of supplementation to significantly improve clinical outcomes. It is also important to determine the use of the acids on different stages of development. n-3 LCPUFAs represent an attractive supplementary alternative, because they are perceived as a natural substance rather than an artificial supplement. In sum, the dietary consumption and supplementation of n-3 LCPUFAs could provide physical and mental health benefits for development.
